# Individual differences in explicit and implicit visuomotor learning and working memory capacity

**DOI:** 10.1038/srep36633

**Published:** 2016-11-08

**Authors:** Antonios I. Christou, R. Chris Miall, Fiona McNab, Joseph M. Galea

**Affiliations:** 1 School of Psychology, University of Birmingham, UK; 2 School of Applied Social Sciences, De Montfort University, UK; 3 Department of Psychology, University of York, UK

## Abstract

The theoretical basis for the association between high working memory capacity (WMC) and enhanced visuomotor adaptation is unknown. Visuomotor adaptation involves interplay between explicit and implicit systems. We examined whether the positive association between adaptation and WMC is specific to the explicit component of adaptation. Experiment 1 replicated the positive correlation between WMC and adaptation, but revealed this was specific to the explicit component of adaptation, and apparently driven by a sub-group of participants who did not show any explicit adaptation in the correct direction. A negative correlation was observed between WMC and implicit learning. Experiments 2 and 3 showed that when the task restricted the development of an explicit strategy, high WMC was no longer associated with enhanced adaptation. This work reveals that the benefit of high WMC is specifically linked to an individual’s capacity to use an explicit strategy. It also reveals an important contribution of individual differences in determining how adaptation is performed.

The ability to retain information for a short period of time, working memory (WM), is known to support many cognitive functions^1^,^2^. Interestingly, an association is also seen with motor function. For example, WM capacity (WMC) predicts learning rate during the early stage of a visuomotor adaptation task, with significant overlap in the pattern of neural activation associated with spatial WM and early adaptation^3^,^4^. Although this work suggests that early, but not late, visuomotor adaptation engages WM processes, it fails to explain this difference.

The literature emphasises the role of implicit learning in visuomotor adaptation tasks. For instance, when an explicit aiming strategy is given to participants; it is quickly overridden by implicit learning^5^. In addition, studies of amnesic patients emphasised that motor learning does not rely on declarative memory^6^,^7^. However, more recent studies have suggested an important interplay between explicit and implicit learning. This was indirectly supported by modelling^8^ and inferring implicit versus explicit learning by the after effect and catch trials, respectively^9^. Direct evidence was provided by Taylor, Krakauer & Ivry^10^ who introduced a novel reaching paradigm in which the contribution of explicit learning was estimated from participant’s report of their aiming direction, while the contribution of implicit learning was estimated by the difference between the intended aiming angle and their movement angle. It was shown that explicit learning, driven by the error from the target, involved initially large explorations of aiming direction biased towards the correct solution, whereas implicit learning, driven by sensory-prediction error, was slow and monotonic^10^.

Although the cerebellum plays a key role in visuomotor learning^11^, activation of the prefrontal cortex (PFC) and specifically the dorsolateral PFC (DLPFC) has been consistently observed during adaptation^3^,^12^. It has been suggested that the PFC is directly associated with the employment of strategic processes^13^. Given the relationship between WM and PFC^14^, this raises the possibility that the link between WM and adaptation is specific to the engagement of an explicit strategic component. However, we have previously shown that even when participants are unaware of the perturbation, so that implicit adaptation must dominate, a cognitively demanding secondary task can still disrupt adaptation^15^. This suggests that implicit adaptation requires cognitive resources and supports an alternative hypothesis in which the positive association with WM extends to implicit adaptation. A third possibility is that those with low WMC show only a small contribution of explicit adaptation, but compensate with enhanced implicit adaptation. For example, within an individual, the systems may operate in parallel or in a “push-pull” manner^10^. Our individual differences approach enabled us to investigate the way in which WMC predicts the contribution of implicit and explicit adaptation systems between individuals.

Adopting the paradigm introduced by Taylor *et al*.^10^, we obtained measures of explicit and implicit learning during visuomotor adaptation, as well as WM capacity for each participant. We hypothesized that WMC would predict the contribution of explicit adaptation, and their association would be positive. We predicted that a positive correlation would not be seen between WMC and implicit adaptation. Furthermore, we predicted that in a paradigm in which explicit learning was minimised, we would eliminate any positive association with WMC. This would indicate that despite being challenged with the same task, individuals may differ in the way they adapt, with explicit strategy making a greater contribution for those with high WMC.

## Results

**Figure 1:**
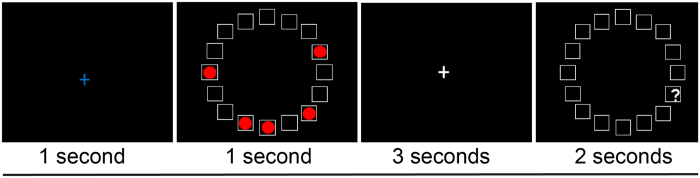
Spatial WM task. Following a fixation period of 1 second, participants were asked to remember the positions of three, four, five or six red target circles presented simultaneously in a circular grid. Following a 3 second delay, a question mark (probe) appeared in or adjacent to one of the target positions. Participants were asked to make a button press with the index or middle finger of their right hand, to indicate whether a circle had appeared at the location indicated.

For each participant, a computer-based visual-spatial WM task was used to obtain a measure of WMC^16^. Participants were asked to remember the positions of three, four, five or six red circles (targets) presented simultaneously for 1 second in a circular array (Fig. 1). Participants were asked to make a button press to indicate whether a probed location corresponded to a target position. WMC was estimated with the K-value, estimating how much information can be stored in WM^17^.

**Figure 2:**
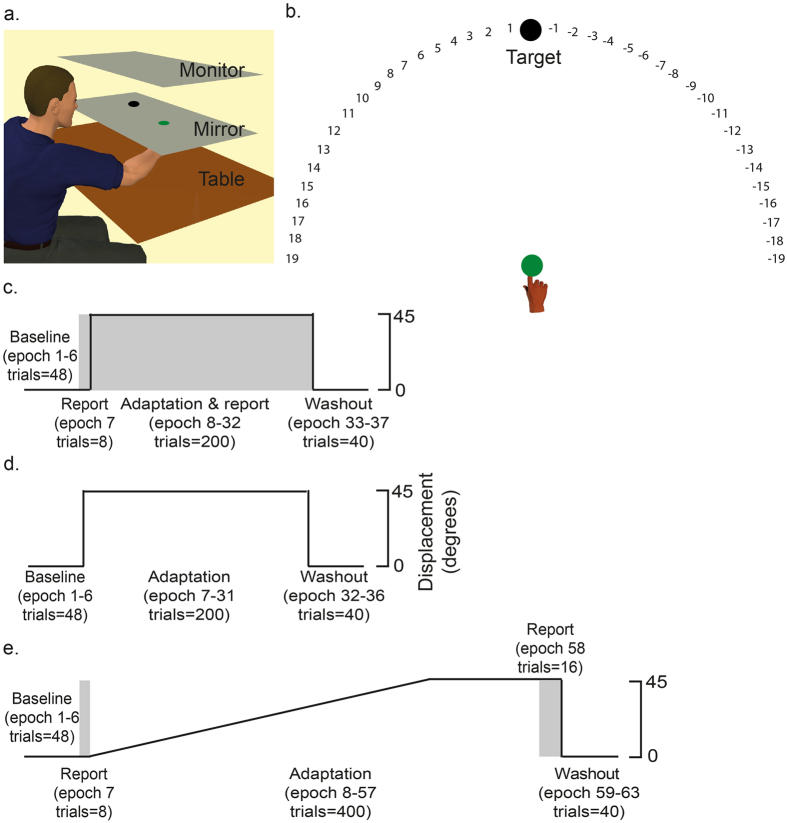
Visuomotor adaptation task. (**a**) Experimental set up. Participants made reaching movements towards targets displayed on a screen. (**b**) Verbal report task (Taylor *et al*.^10^). The target was placed at 0 with numbers being placed either side of the target at 5° intervals. Participants were asked to report which number they were planning to move their finger towards. This was used as a direct measure of participant’s trial-by-trial cognitive strategy. (**c**) Experiment 1: participants adapted to an abrupt 45° visuomotor rotation with terminal feedback whilst performing the verbal report task (grey) prior to each movement. (**d**) Experiment 2: participants adapted to an abrupt 45° visuomotor rotation with terminal feedback. (**e**) Experiment 3: participants adapted to a gradual 45° visuomotor rotation with online feedback. The verbal report task (grey) was performed at the start and end of the adaptation block. Amount of trials and epoch number are provided in brackets. Between baseline, adaptation and washout there were short rest periods.

For each participant, the WMC task was followed by a visuomotor adaptation task. Three different visuomotor tasks were employed (Experiments 1–3), with each person participating in only one experiment. For all participants, their right index finger was attached to a Polhemus motion tracking system underneath a horizontally suspended mirror. The mirror prevented direct vision of the hand and arm, but showed a reflection of a computer monitor mounted above that appeared to be in the same plane as the hand (Fig. 2a). For all experiments, a target was displayed in one of eight positions arrayed radially 45° apart at 7 cm from a central starting box and a cursor represented the position of the participant’s index finger^18^.

For each trial, participants were required to make a fast, “shooting” movement through the target, from the starting position. Angular hand direction (°) was calculated as the difference between the angular hand position and angular target position at the point when the cursor intersected an invisible circle with a radius of 7 cm which was centred on the starting position. Positive values indicated CW error whereas negative values indicated CCW error. During veridical feedback, the goal was for reach direction error to be 0°. However with a visuomotor transformation, hand direction had to compensate; that is, for a −45° (CCW) visuomotor rotation, a hand direction of +45° (CW) was required for the cursor to hit the target. When the visuomotor transformation was removed (washout), we did not give any instruction to stop aiming.

In order to test the association between WMC to adaptation success, we used absolute values of the difference in angle from 45°. This disregards the direction of movement relative to the target, and considers only the extent to which the angle differs from the target angle. For illustration we show the angle that resulted from subtracting the angle of movement from the target angle, but for the statistical analyses we use absolute values as we were interested in adaptation success, and not whether a participant moved or aimed to the right or the left of the target. Using the raw values did not affect the results; the same correlations were statistically significant, irrespective of the measure of adaptation.

For analysis, epochs were created by binning 8 consecutive movements (including 1 movement towards each target). We calculated performance averages across the last 2/3 of epochs within each block for each of the 3 experiments. In addition, reaction time (RT: difference between target appearing and participant moving out of the start box) and movement time (MT: difference between reaction time and movement end) were calculated for each trial.

### Experiment 1: explicit and implicit visuomotor adaptation

Experiment 1 employed a task that was adapted from Taylor *et al*.^10^ and enabled the dissociation of explicit and implicit processes during visuomotor adaptation (Fig. 2b). Participants completed 4 blocks with terminal feedback (Fig. 2c).

During Block 1 (baseline), which involved veridical vision, all participants (n = 30) showed similar accurate reaching behaviour, with there being no significant correlation between WMC and hand direction (r = −0.19, p > 0.25, r^2^ = 0.03), RT (r  = −0.17, p > 0.25, r^2^ = 0.03), or MT (r = −0.30, p = 0.10, r^2^ = 0.04) (Table 1). Block 2 (baseline report) involved veridical vision and prior to commencing each trial participants were required to verbally report the direction in which they were aiming to move their finger (explicit aiming strategy). In these trials, the target was presented at the centre of a semi-circular arc of numbers (Fig. 2b). Participants were asked to report which number they were planning to move their finger towards^10^,^19^,^20^. Once they had provided this verbal response, the numbers disappeared and the participants performed the reaching movement. This reporting block was used simply to introduce the participants to the reporting procedure.

**Figure 3:**
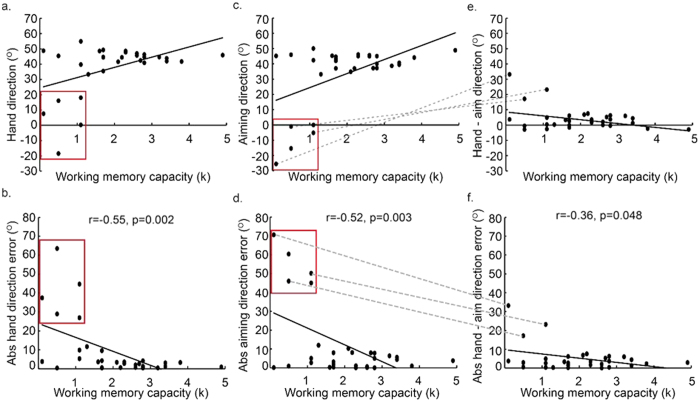
Experiment 1: explicit and implicit visuomotor adaptation. (**a**,**b**) The significant correlation between WMC and hand direction (**a**) and between WMC and absolute hand direction error (**b**) during adaptation to an abrupt 45° visuomotor rotation. (**c**,**d**) The significant correlation between WMC and aiming direction, our measure of explicit adaptation derived from the verbal report task (**c**), and between absolute aiming direction error and WMC (**d**). (**e**,**f**) The significant correlation between WMC and hand direction minus aim direction (**e**), our measure of implicit adaptation, and between WMC and absolute values of hand direction minus aim direction (**f**). For illustration we show the angle that resulted from subtracting the angle of movement from the target angle (**a**,**c**,**e**), but for the statistical analyses we use absolute values representing the angle by which the implicit adaptation differed from 45° (**b**,**d**) or 0° (**f**). Using the absolute values as opposed to the raw values for the angles did not affect the results; the same correlations were statistically significant, irrespective of the measure of adaptation. The red rectangles show the data-points from the five participants who did not show any explicit adaptation in the direction of the target, and who drove the correlation. The dashed grey lines show that three of the participants who did not show any explicit adaptation in the direction of the target also showed high implicit adaptation.

In Block 3 (adaptation and report), a 45° CCW visuomotor rotation of the cursor, relative to the hand, was imposed. The participants were required to verbally report their explicit aiming strategy (angle) prior to each movement. When exposed to the 45° rotation, there was a negative correlation between WMC and absolute hand direction error (r = −0.55, p = 0.002, r^2^ = 0.30, Fig. 3a,b), showing that those with greater WMC were more successful at the adaptation task. As well as testing the linear association between WMC and absolute hand error (HE = β*WMC + α, where α is the intercept, β is the regression coefficient and HE is absolute hand direction error), we also tested an exponential association between WMC and absolute hand direction error (HE = exp^β(WMC)^ + α). Each model explained a significant amount of variance, with the linear model giving a slightly better fit (linear model: α = 23.89, β = −7.30, p = 0.002, r^2^ = 0.30; exponential model: α = 13.00, β  = −0.70, p = 0.002, r^2^ = 0.29). The fact that the exponential model did not explain more variance than the linear model is likely to be due to the fact that the association is driven by five participants who show poor adaptation, so that the participants appear to fall into two groups. This was explored further with additional analyses (see below). RT (r = 0.20, p > 0.25, r^2^ = 0.04; Table 1) and MT (r = −0.32, p = 0.09, r^2^ = 0.1; Table 1) were not significantly associated with WMC.

On each trial, participants were required to report the direction to which they were aiming, before moving (explicit aiming strategy). There was a significant negative correlation between WMC and absolute aiming direction error (r = −0.52, p = 0.003, r^2^ = 0.27; Fig. 3c,d). As shown by Fig. 3c/d, five participants either failed to show any explicit adaptation and aimed towards the target despite the rotation, or aimed in the opposite direction.

By subtracting aiming direction from hand direction, we were able to estimate the amount of implicit adaptation on each trial^10^. There was surprisingly little implicit adaptation, but a significant negative correlation between WMC and absolute values of implicit adaptation (r = −0.36, p = 0.048, r^2^ = 0.13; Fig. 3e,f). As shown by Fig. 3e/f, there were large values for implicit adaptation in the correct direction (ie. compensating for the 45° rotation) by participants with low WMC.

Block 4 (washout) involved veridical vision, and no report was required. There was only a small ‘after-effect’, which did not significantly correlate with WMC (r = 0.17, p = 0.37).

### Participants showing no explicit adaptation in the correct direction

From Fig. 3, it is clear that the observed correlations between adaptation (in Block 3) and WMC seem to be driven by five participants (the data-points within the red squares in Fig. 3) who showed either no explicit adaptation, or explicit adaptation in the wrong direction. When data from these five participants were removed from the analysis, none of the correlations were statistically significant (the correlation between absolute hand direction error and WMC: r = −0.39, p = 0.06, r^2^ = 0.15; the correlation between absolute aiming direction error and WMC: r = 0.09, p > 0.25, r^2^ = 0.007; the correlation between absolute hand - aim direction error and WMC: r = 0.09, p > 0.25, r^2^ = 0.007). It is not clear whether these five participants did not have the cognitive resources to successfully employ an explicit strategy, or were not sufficiently motivated to do so. As shown by Fig. 3, there were two participants with a WMC of less than 2 who did show explicit adaptation in the correct direction.

Although further work is needed to understand the basis for a failure to show successful explicit adaptation, our data do suggest that WMC does not predict adaptation success throughout the range of WMC scores obtained. Our group was better characterised as those who did and those who did not show successful explicit adaptation. This result highlights the need for caution when associating WMC with adaptation and suggests that it is particularly important to include a report task, so that participants who do not explicitly aim in the correct direction can be identified.

**Figure 4:**
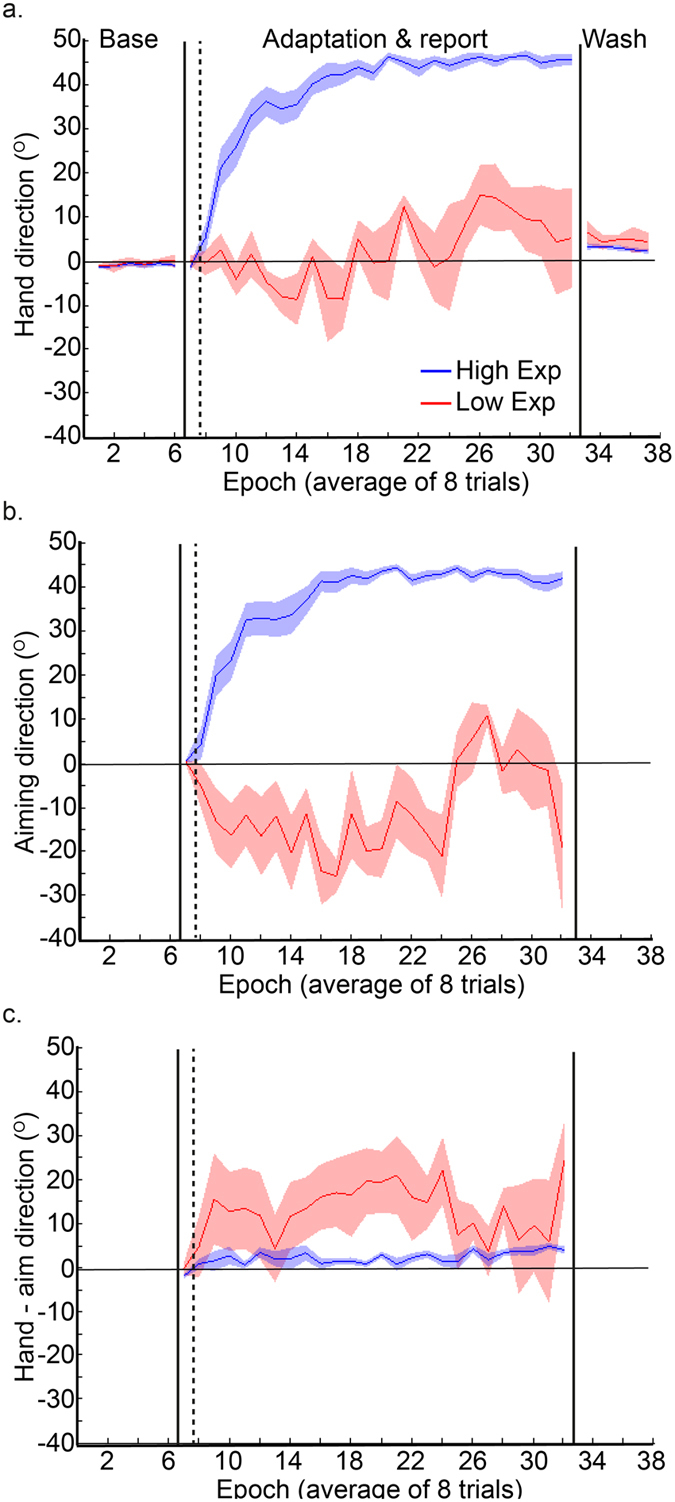
Experiment 1: participants who did and did not show explicit adaptation in the direction of the target. Hand direction group data (line = group mean, shaded area = standard error of mean across group) during adaptation to an abrupt 45° visuomotor rotation. The “High Exp” (n = 25, blue) group showed explicit adaptation (and aim angle of >0 in the direction of the target). The “Low Exp” (n = 5, red) group did not show any explicit adaptation in the direction of the target (they are the group within the red rectangles in Fig. 3). (**a**) The “High Exp” group showed greater adaptation overall. (**b**) Aiming direction (°) derived from the verbal report task for the two groups. (**c**) The “Low Exp” group showed greater implicit adaptation.

Interestingly, as indicated by the grey dashed lines in Fig. 3, three of the five participants who did not show any explicit adaptation in the correct direction, had the highest values for implicit adaptation, suggesting that explicit and implicit adaptation may be operating in a push-pull manner between individuals. Figure 4 reveals that the five participants who did not show any explicit adaptation (red curves, the low explicit adaptation group) showed greater implicit adaptation in the direction of the target, throughout the adaptation period, relative to the other participants (blue curve, high explicit adaptation group).

### Experiments 2 and 3

With Experiment 1 we observed a positive association between WMC and explicit adaptation using a task in which participants reported their direction of aim. In contrast there was no positive correlation between WMC and implicit adaptation; in fact we observed a negative correlation. This suggests that the benefit of high WMC for adaptation^3^,^4^,^21^ is specific to the explicit component. With Experiments 2 and 3 we sought to verify this conclusion with a paradigm that did not involve the participants reporting their aiming direction during adaptation. We predicted that there should not be a positive association between WMC and adaptation in situations where the task does not promote an explicit strategy. Experiment 2 used a simple visuomotor rotation task in which participants adapted to an abrupt 45° displacement with terminal visual feedback (Fig. 2d). An abrupt rotation with terminal feedback should promote an explicit strategy^10^,^20^. In contrast, Experiment 3 involved adaptation to a gradual 45° rotation with online visual feedback (Fig. 2e), and thus did not encourage the use of an explicit strategy.

### Experiment 2: abrupt visuomotor adaptation

**Figure 5:**
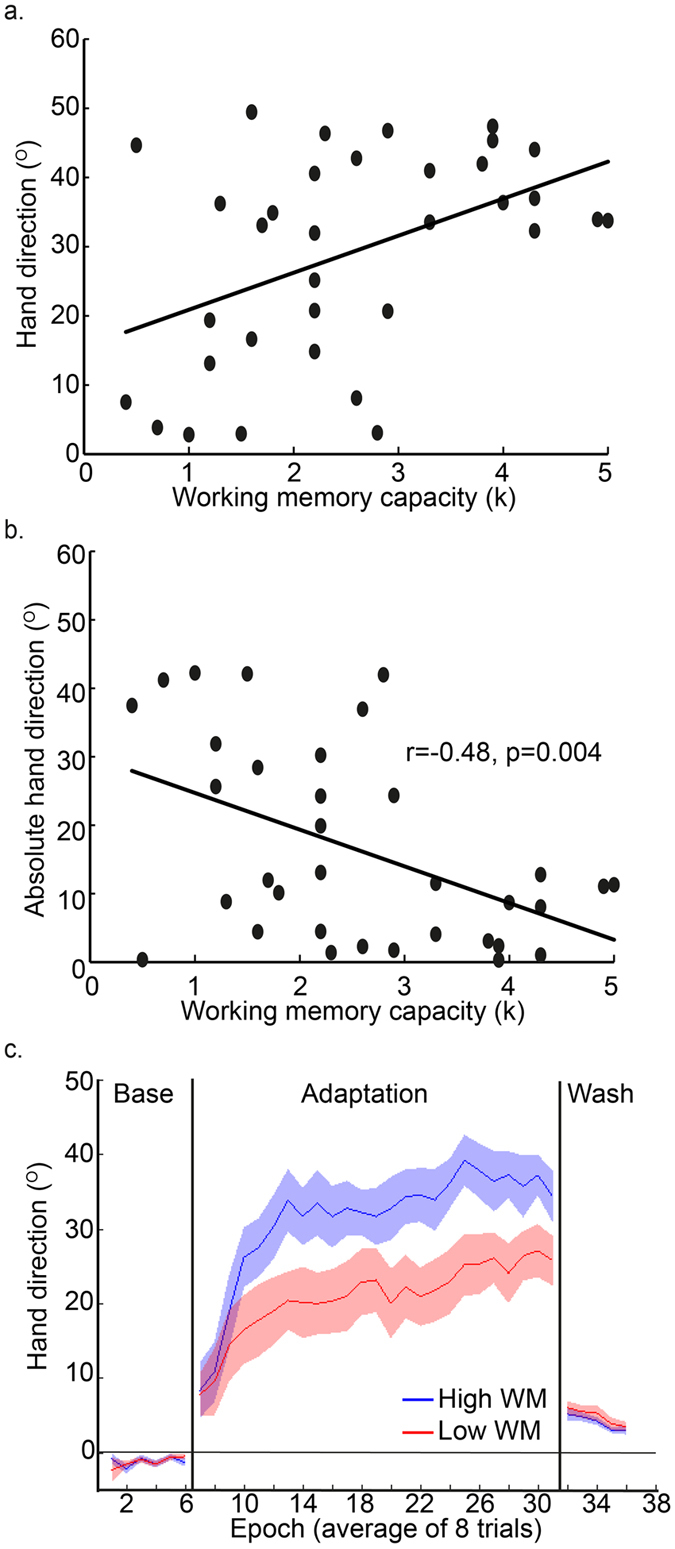
Experiment 2: Abrupt adaptation. (**a**,**b**) The significant correlation between WMC and hand direction (**a**) and between WMC and absolute hand direction error (**b**) during adaptation to an abrupt 45° visuomotor rotation when participants did not report their hand direction. (**c**) For illustration we performed a median split, separating the group into those with high and low WMC. Hand direction throughout the experiment is shown for the high WMC (n = 17, blue) and low WMC (n = 17, red) groups (line = group mean, shaded area = standard error of mean across group). The high WMC group showed greater adaptation relative to the low WM group, however no differences were observed during baseline or washout.

For Experiment 2, during baseline with veridical vision, all participants (n = 34) showed similar accurate reaching behaviour, with there being no significant correlation between WMC and hand direction (r = −0.05, p > 0.25, r^2^ = 0.003), RT (r = 0.03, p > 0.25, r^2^ = 0.001) or MT (r = −0.08, p > 0.25, r^2^ = 0.007) (Table 1). However following exposure to a sudden 45° rotation (Block 2), a significant correlation was observed between absolute hand direction error and WMC (r = −0.48, p = 0.004, r^2^ = 0.23, Fig. 5a,b), whereby those with high WMC displayed a greater level of adaptation. When we restricted our analysis to ‘early’ adaptation (the first 96 trials, a number similar to Anguera *et al*.^3^), the correlation between WMC and absolute hand direction error was very close to reaching significance (r = −0.34, p = 0.051, r^2^ = 0.11). RT (r = 0.15, p > 0.25, r^2^ = 0.02) and MT (r = −0.29, p = 0.10, r^2^ = 0.08) (Table 1) did not show a significant correlation with WMC during adaptation. When the visual rotation was removed (washout), all participants showed a small ‘after-effect’ whereby their hand direction did not instantly return back to baseline levels (Fig. 5c). Surprisingly, despite the large differences in adaptation, there was no significant association between WMC and hand direction during washout (r = −0.08, p > 0.25, r^2^ = 005). This indicates that high WMC was associated with successful adaptation when the task design (abrupt with terminal feedback) promoted an explicit strategy. For illustrative purposes, we performed a median split to separate the participants into high and low WMC groups. Figure 5c clearly shows that the high WMC group showed a greater amount of adaptation relative to the low WMC group.

### Experiment 3: gradual visuomotor adaptation

**Figure 6:**
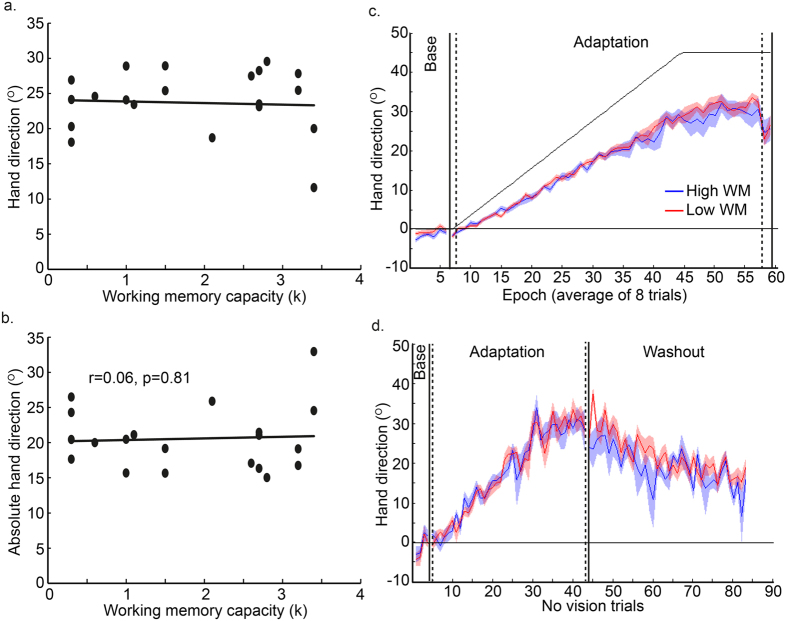
Experiment 3: Gradual adaptation. (**a**,**b**) Neither hand direction (**a**) not absolute hand direction error (**b**) during adaptation to a gradual 45° visuomotor rotation correlated with WMC. (**c**) Again, for illustration, hand direction is shown for the two groups (high and low WMC, based on a median split; line = group mean, shaded area = standard error of mean across group). The high WM (n = 10, blue) and low WM (n = 10, red) group showed similar adaptation. (**d**) No vision trials were used throughout to provide a relatively clean measure of sensorimotor recalibration (implicit process). There were no observable differences between high and low WM groups during adaptation but a suggestion that the low WM group showed a greater ‘after-effect’ during washout.

Experiment 3 involved adaptation to a gradual 45° rotation with online visual feedback, to reduce the likelihood of the participant using an explicit strategy. During baseline, all participants (n = 20) showed similar accurate reaching behaviour, with there being no significant correlation between WMC and hand direction (r = 0.31, p = 0.18, r^2^ = 0.10), between WMC and RT (r = 0.40, p = 0.08, r^2^ = 0.16) or between WMC and MT (r = −0.03, p > 0.25, r^2^ = 0.001) (Table 1). Participants were then gradually exposed to a 45° rotation over the next 300 trials, with it then remaining constant for a further 116 trials. Although it is clear that participants did not fully adapt to the gradual visual rotation, there was no significant correlation between absolute hand direction error and WMC (r = 0.06, p > 0.25, r^2^ = 0.003; Fig. 6a–c).

As Experiment 3 involved fewer participants than Experiment 1 or 2, we wanted to ensure the lack of significance was not simply an issue of statistical power. On the basis of the r^2^ values from the correlations (Experiment 1: 0.30, Experiment 2: 0.23), we estimated (G*Power; http://www.gpower.hhu.de/en.html) that power of 0.93 and 0.87 was achieved in Experiment 1 and 2, respectively. For Experiment 3 (r^2^ = 0.003), a total sample size of 3168 would be required to achieve similar power of 0.87. This indicates that the effect size was negligible and adding an additional 14 participants, making the sample size similar to Experiment 2, would have made little difference.

To ensure the participants were indeed unaware of the perturbation, participants were asked to report their aiming direction at the beginning and end of the adaptation block^10^. For each time point, only two participants reported a non-zero aiming angle, and these were <5°. This suggests that by the end of the adaptation block, participants had not developed an explicit strategy. To examine behaviour further we had included no vision trials throughout baseline, adaptation and washout. We surmised that no vision trials during washout would give a better indication of the level of implicit adaptation achieved. Similarly to the vision trials, we observed no significant association between WMC and hand direction (r = 0.14, p > 0.25, r^2^ = 0.02). During washout, there was a slow drift back to baseline performance (Fig. 6d). Although not significant, there is a suggestion that participants with low WM showed a greater ‘after-effect’ than participants with a high WM (r = −0.40, p = 0.08, r^2^ = 0.16). Once again for illustrative purposes, we median split the participants into high and low WMC groups. Figure 6c,d clearly shows that the high and low WMC groups displayed a similar amount of adaptation. Although the figure suggests that the low WM group showed a greater after-effect, this did not reach statistical significance (t (18) = 1.60, p = 0.13).

### Comparison between Experiments 2 and 3

There was a significant difference in the correlation between WMC and absolute direction hand error during adaptation for Experiments 2 and 3 (Fisher’s Z = −1.93, p = 0.03, Cohen’s q = 0.46, one tailed-test). As predicted, there was an association between WMC and absolute hand direction error only when the rotation was applied abruptly (Experiment 2), and not when it was introduced gradually, restricting the involvement of explicit processes (Experiment 3).

## Discussion

### Summary

This study examined whether the link between WMC and visuomotor adaptation is specific to the use of an explicit strategy, or extends to implicit adaptation. First, we replicated previous findings linking high WMC with more successful adaptation, during a standard visuomotor rotation task. By obtaining separate measures of explicit and implicit adaptation, we found that this association was driven by five participants who failed to show any explicit adaptation in the correct direction. Interestingly, a majority of these five participants also displayed a high level of implicit adaptation, which reduced their hand error. We also showed that when adaptation was dominated by implicit learning, high WMC was no longer associated with enhanced adaptation.

### Explicit and Implicit adaptation and the association with WMC

Experiments 1 and 2 replicated previous work showing a correlation between spatial WMC and adaptation^3^, although in their study this was specific to “early” adaptation. We extended the approach to separately consider two components of adaptation: a cerebellar-dependent implicit process, that involves a sensory-prediction error signal used to update a forward model, and a non-cerebellar explicit process in which participants implement a strategy.

DLPFC activity, which may be associated with WM, has been observed during adaptation tasks^3^,^12^, and may be specifically associated with the employment of strategic processes^13^. Furthermore, a specific impairment shown by older adults during ‘early’ adaptation to an abrupt perturbation, but not to a gradual perturbation^22^,^23^, appears to be due to an inability to engage DLPFC-dependent spatial WM^13^,^22^,^24^. This work raises the possibility that the link between WMC and adaptation is specific to the implementation of a cognitive strategy.

An alternative account would be that both explicit and implicit adaptation show a positive association with WMC. We have previously shown that even when participants are unaware of the perturbation, so that implicit adaptation must dominate, a cognitively demanding secondary task can still disrupt adaptation^15^. This suggests that implicit adaptation requires cognitive resources and that the association with WMC extends to implicit adaptation. A third alternative is that explicit adaptation shows a positive association with WMC, but that implicit adaptation shows a negative association with WMC, in line with implicit and explicit systems operating in a push-pull manner.

By employing the paradigm of Taylor, Krakauer & Ivry^10^, and separately measuring explicit and implicit adaptation, we observed, for the first time, a *positive* association between WMC and explicit adaptation, but a *negative* association between WMC and implicit adaptation, supporting the third account. We were also able to identify five participants who failed to show any explicit adaptation in the correct direction. When excluding data from these participants, we no longer observed a significant association between WMC and adaptation. This suggests that the involvement of WMC in explicit adaptation could be “all or none”, whereby a minimum WMC is needed for strategy development, but extra capacity gives no additional benefit. Alternatively, the five participants who failed to show any explicit adaptation in the correct direction may have not been sufficiently motivated to perform the experiment correctly. Irrespective of the cause, these results suggest that previously reported correlations between adaptation and WMC^3^, which did not distinguish between explicit and implicit adaptation, may have been misleading. Enhanced implicit adaptation in those with low WMC and little explicit adaptation, could give the impression of a linear association between WMC and adaptation, rather than showing a difference between two groups of participants.

### WMC is not crucial when implicit learning dominates

Explicit and implicit processes have often been compared using adaptation to an abrupt and gradual perturbation^15^,^25^,^26^. The limited movement errors resulting from gradual perturbation are thought to be corrected implicitly. Our previous work has suggested that despite minimal awareness, implicit adaptation can be disrupted by a cognitively demanding secondary task^15^. At the beginning and end of the gradual adaptation period of Experiment 3 we found no evidence of any participants using a strategy. Critically, there was no significant association between adaptation and WMC, unlike in Experiment 2 where explicit adaptation was encouraged by an abrupt rotation. This supports our finding from Experiment 1 that the positive association between WMC and adaptation is directly linked to the ability to use an explicit strategy.

We are not suggesting there is a categorical difference between abrupt and gradual adaptation. We believe, along with others, that there is a continuum for the contribution of implicit and explicit learning processes, which can be affected by task parameters, including the size of the perturbation, the number of trials, target position, visual feedback conditions etc^15^,^20^,^25^,^27^. In our study, we pragmatically chose two extremes to highlight the specificity of the association with WMC.

### WMC and explicit adaptation

Our study is the first to directly link WMC with the adoption of an explicit strategy during visuomotor adaptation. Our results show that even when task parameters remain the same, individual differences between participants, such as WMC, predict the manner in which they adapt. These findings may inform rehabilitation. While focussing on implicit processes may be more effective for motor rehabilitation following stroke, for tasks where an explicit strategy is beneficial, WM training^28^ could provide an effective adjunct treatment.

The nature of the association we see between WMC and strategic visuomotor adaptation remains to be clarified. Most obviously, a higher WMC, or a greater ability to control attention, may better equip an individual to maintain different hypotheses and action-response associations during the execution of a cognitive strategy. A related possibility is that participants who more readily use an effective strategy benefit from this approach during both adaptation and WM tasks^29^. However, it has been argued that strategies that can be used for WM tasks tend to be task-specific and simple, and that individual differences in strategic behaviour cannot explain the link between WMC and, for example, reasoning ability^30^. Another possibility is that a lack of motivation to perform the task correctly affects measures of WMC and explicit adaptation, but not implicit adaptation. Our finding of high implicit adaptation in participants who failed to show explicit adaptation in the correct direction provides some support for this account.

### The association between explicit and implicit adaptation

Our finding of a significant *positive* correlation between WMC and explicit adaptation, but a significant *negative* correlation between WMC and implicit adaptation suggests that implicit and explicit systems operate in a “push-pull” manner, between individuals. For other types of motor learning, such as sequence learning^31^,^32^,^33^, restricting the contribution of explicit learning, enhances implicit learning. As far as we are aware, our study is the first to suggest a “push-pull” association between explicit and implicit learning between individuals, rather than between experimental conditions. It suggests that for visuomotor adaptation enhanced implicit adaptation may compensate in individuals who show less explicit adaptation. This indicates that implicit adaptation processes may not be monotonic, as previously suggested^13^. An alternative account would be that those with high implicit adaptation, who tend to have a low WMC, have smaller aiming angles (or aim in the wrong direction) as they are relying on implicit adaptation. However, our significant correlation between WMC and overall adaptation indicates that those with low WMC do not employ sufficient explicit adaptation to bring them up to the same level of overall adaptation as those with higher WMC.

### Conclusion

In conclusion, we have shown that the benefit of high WMC during visuomotor adaptation is associated with the ability to implement an effective aiming strategy. When awareness of the perturbation was reduced, and implicit learning dominated, WMC no longer predicted the level of adaptation achieved. We also observed that whereas lower WMC predicted less explicit adaptation, it predicted greater implicit adaptation, suggesting a “push-pull” interaction between explicit and implicit adaptation, between individuals, and highlighting an important contribution of individual differences in determining the relative contribution of implicit and explicit processes to learning. This promises to open a new chapter in motor learning research where individual differences, such as memory capacity, personality traits and even genetics, predict the relative contributions of different processes to learning.

## Methods

### Participants

96 self-reported, right-handed individuals with no history of neurological or psychiatric conditions (males: 14, mean age: 19.27 years, SD: 1.9) participated in the study. The study was approved by the Ethical Review Committee of the University of Birmingham and was in accordance with the Declaration of Helsinki. Written, informed consent was obtained from all participants. 36 performed Experiment 1, and data from 6 participants were excluded as they had a WMC score of < 0 (a score of below zero suggested participants were not paying attention to the task, leading them to performing it incorrectly and at chance level) leaving data from 30 participants (mean age: 19.33 years, SD: 1.3). For Experiment 2, data were collected from 38 participants, and data from 4 participants were excluded as they had a WMC score of < 0, leaving data from 34 participants (mean age: 18.88 years, SD: 0.98). 22 participants performed Experiment 3, and data from 2 participants were excluded as they had a WMC score of < 0, leaving data from 20 participants (mean age: 19.85 years, SD: 2.9). No statistical methods were used to predetermine the sample size for each experiment with funding/time restraints being the principal rule for stopping collection. Although this led to variable participant numbers across the 3 experiments, posthoc power analysis was used in the results section to show that this did not affect our conclusions. All participants reported that they were not taking any medication and had a normal amount of sleep the previous night. Participants were recruited through online advertising and posters, and received money (£6) or study credits as compensation upon completion of the study.

### Experimental tasks

Each participant completed a WMC task, followed by a visuomotor adaptation task. Three different visuomotor tasks were employed (Experiments 1–3), with each person participating in only one experiment.

#### Working Memory Capacity (WMC) task

A computer-based visual-spatial WM task was used to obtain a measure of WMC^16^. Participants were asked to remember the positions of three, four, five or six red circles (targets) presented simultaneously for 1 second in a circular array (Fig. 1). Following a 3 second delay a question mark (probe) appeared in one of the target cells or an adjacent cell in the array. Participants were asked to make a button press with the index or middle finger of their right hand, to indicate whether the probed location corresponded to a target position. There were 10 trials of each array size, half of which required a “yes” response.

#### Visuomotor task

The three experiments shared most details of their design; differences will be described below. In all three, participants were seated with their forehead supported on a headrest in front of a visual workstation. Their semipronated right index finger was attached to a Polhemus motion tracking system (Colchester, VT, USA) underneath a horizontally suspended mirror. The mirror prevented direct vision of the hand and arm, but showed a reflection of a computer monitor (30-inch; 1280 × 1024 pixel resolution) mounted above that appeared to be in the same plane as the hand (Fig. 2a). The visual display consisted of a 1 cm-diameter starting box, a green cursor (0.25 cm diameter) representing the position of their index finger, and a circular white target (0.33 cm diameter). For all experiments, a target was displayed in one of eight positions arrayed radially 45° apart at 7 cm from the central starting box^18^. At the beginning of each trial, participants were asked to move their index finger to the start position, which was located at the centre of the screen, and a target then appeared. Participants were required to make a fast, “shooting” movement through the target, such that online corrections were effectively prevented. At the moment the cursor passed through the invisible boundary circle (an invisible circle centred on the starting position with a 7 cm radius), the cursor was hidden and the intersection point was marked with a yellow square to denote the terminal (endpoint) error. In addition, a small square icon at the top of the screen (15 cm above the start position) changed colour based on movement speed. If the movement was completed within 100–500 msec, then it remained white. If the movement was slower than 500 msec, then the box turned red (too slow). Note, no feedback was given regarding reaction time. After each trial, subjects moved back to the start. The cursor indicating their hand position only reappeared when they were within 2 cm of the central position. For all experiments, the targets were presented pseudo-randomly so that every set of eight consecutive trials included all eight target positions (1 epoch).

### Experimental protocol

#### Experiment 1: explicit and implicit visuomotor adaptation

Experiment 1 replicated the task developed by Taylor *et al*.^10^ to directly assess the contribution of explicit and implicit learning to visuomotor adaptation. Participants completed 4 blocks with terminal feedback. Block 1 (baseline) involved 48 trials with veridical vision. Block 2 (baseline report) involved 8 trials with veridical vision: prior to commencing each trial participants were required to verbally report the direction in which they were aiming to move their finger (explicit aiming strategy). In these trials, the target was presented at the centre of a semi-circular arc of numbers displayed at 5° intervals (Fig. 2b). Clockwise (CW) of the target were negative numbers from 1–19, and Counter-CW (CCW) of the target were positive numbers from 1–19. Participants were asked to report which number they were planning to move their finger towards^10^,^19^,^20^. Once they had provided this verbal response, the numbers disappeared and the participants performed the reaching movement. This reporting block was used to introduce the participants to the reporting procedure. Block 3 (adaptation and report) involved 200 trials where a 45° CCW visuomotor rotation of the cursor, relative to the hand, was imposed. The participants were required to verbally report their explicit aiming strategy (angle) prior to each movement. Block 4 (washout) involved 40 trials with veridical vision, and no report was required. This experiment is described by Fig. 2c.

#### Experiment 2: abrupt visuomotor adaptation

Experiment 2 involved a simple abrupt visuomotor adaptation task in which participants were not required to report their explicit aiming strategy (Fig. 2d). To this end, participants completed 3 blocks with terminal error feedback (i.e. participants were not provided with online vision of their movement). Block 1 (baseline) involved 48 trials with veridical vision (epoch 1–6). Block 2 (adaptation) involved 200 trials where a 45° counter clockwise (CCW) visuomotor rotation of the cursor was imposed relative to the hand (epoch 7–31). Block 3 (washout) involved 40 trials with veridical vision (epoch 32–36).

#### Experiment 3: gradual visuomotor adaptation

Experiment 3 was designed to present a visuomotor task where the implementation of an explicit strategy would be minimised (Fig. 2e). Participants completed 3 blocks with both online vision of their movement and terminal feedback. Online feedback of the finger position was provided by the cursor during the movement as this is thought to optimise error-based/implicit learning^15^,^27^. However, for every 10^th^ movement we removed both online and terminal feedback so that participants made reaching movements without vision; they saw a target but received no feedback as to their movement accuracy (no vision trials). Block 1 (baseline) involved 44 trials with veridical vision and 4 no vision trials. Block 2 (baseline report) involved 8 trials with veridical vision; the participants were required to verbally report their explicit (planned) aiming direction prior to each movement. Block 3 (adaptation) involved 416 trials in which a 45° CCW visuomotor rotation was gradually applied; 39 of these were no vision trials. The rotation began at 0.15° on the first trial of the block and increased by 0.15° on each subsequent trial until it reached a maximum of 45° (trial 300). The 45° rotation was then maintained for the remaining 116 trials. For the last 16 trials of this block, participants were required to verbally report their planned aiming direction prior to each movement. This provided a measure of each participant’s explicit aiming strategy at the end of adaptation. Finally block 4 (washout) involved 40 trials with no vision. Note, this is different to Experiments 1 and 2 where participants had vision during washout. No vision trials were used during this experiment to determine whether WMC influenced the retention of a visuomotor rotation learnt primarily through implicit processes.

### Data analysis

#### Working memory

WMC was estimated with the K-value, estimating how much information can be stored in WM, using a standard formula: *K* = *S (H* − *F*), where *S* is the array size, *H* is the observed hit rate and *F* is the false alarm rate^17^. This formula uses the false alarm rate to correct for guessing and assumes that if *K* items can be held in WM, from an array of *S* items, the probed item would have been one of those held in memory on *K*/*S* of trials, so that performance will be correct on *K*/*S* of the trials. For each participant, the mean *K* of array sizes 5 and 6 was used as our measure of WMC. For inclusion in the study, participants were required to have a K value greater than 0.

#### Visuomotor tasks

Index finger (hand) position (x,y) was collected at 60 Hz using Matlab (The MathWorks, Natick, USA) and the Psychophysics toolbox (http://www.psychtoolbox.org). For each trial, angular hand direction (°) was calculated as the difference between the angular hand position and angular target position at the point when the cursor intersected the 7-cm invisible circle centred on the starting position relative to the central starting point. Positive values indicated CW error whereas negative values indicated CCW error. During veridical feedback, the goal was for reach direction error to be 0°. However with a visuomotor transformation, hand direction had to compensate; that is, for a −45° (CCW) visuomotor rotation, a hand direction of +45° (CW) was required for the cursor to hit the target. In addition, reaction time (RT: difference between target appearing and participant moving out of the start box) and movement time (MT: difference between reaction time and movement end) were calculated for each trial. For all experiments, we removed any trial in which reach direction exceeded 60°^34^. Across experiments, this accounted for less than 4% of trials.

For analysis, epochs were created by binning 8 consecutive movements (including 1 movement towards each target). We calculated performance averages across the last 2/3 of epochs within each block for each of the 3 experiments. It was thought that this provided a good representation of final baseline, final adaptation and final washout (retention) values where performance had plateaued. Specifically for Experiment 1, explicit learning (aiming direction) was defined as the participant’s verbal report of the numbered landmark they were aiming to, multiplied by the spacing of the numbered landmarks (5°). Implicit learning (hand - aim direction) was computed by subtracting the aiming angle from the angular hand direction for each trial^10^,^19^,^20^. For hand direction, averages were calculated for baseline, adaptation and washout blocks. For explicit and implicit learning, an average was calculated for the adaptation block. For Experiment 2, we calculated averages for epochs 3–6 (baseline; 32 trials), 16–32 (adaptation; 136 trials) and 35–37 (washout; 24 trials). For Experiment 3, we first separated the no vision trials (every 10^th^ trial), which were analysed separately: there were 83 no vision trials: 4 in baseline (block 1), 39 in adaptation (block 3; note we did not include no vision trials at the very end of adaptation or during the explicit aiming trials) and 40 in washout (block 4). We calculated averages for baseline (no vision trials 2–4), adaptation (no vision trials 16–43) and washout (no vision trials 57–83). We also calculated averages for the online vision trials; baseline trials (epochs 3–6, 32 trials) and adaptation trials (epochs 24–59, 280 trials).

#### Statistics

All data and statistical analyses were performed using Matlab (The Mathworks, Natwick, MA) and IBM SPSS (IBM Corp. Released 2015. IBM SPSS Statistics for Windows, Version 23.0. Armonk, NY: IBM Corp). Across all 3 experiments we performed Pearson correlations (r) between adaptation performance and WMC. For illustration we show scatter plots of the angle at which the participant moved or aimed, with 45° representing the target angle, against WMC. Angles of <45° resulted in an endpoint that was to CCW to the target (i.e. an “under-shoot”), and angles of >45° resulted in an endpoint that was CW to the target (i.e. an “over-shoot”). However, in order to test the association between WMC to adaptation success, we used absolute values of the difference in angle from 45° as we are interested in whether WMC predicts adaptation success, and not whether a participant moved or aimed to the right or the left of the target. This disregards the direction of movement (absolute hand direction error) or aim (absolute aiming direction error) relative to the target, and considers only the extent to which the angle differs from the target angle. Similarly, for implicit adaptation, for illustration we show the angle that resulted from subtracting the angle of aim from the angle of movement, but for the statistical analyses we use absolute values representing the angle by which the implicit adaptation differed from 0° (absolute hand - aim direction error). Using the absolute values as opposed to the raw values for the angles did not affect the results. The same correlations were statistically significant, irrespective of the measure of adaptation.

The significance level was set at p < 0.05. All correlations were two-tailed, To provide a measure of effect size, we report R-squared (r^2^) values for each correlation.

## Additional Information

**How to cite this article**: Christou, A. I. *et al*. Individual differences in explicit and implicit visuomotor learning and working memory capacity. *Sci. Rep.* **6**, 36633; doi: 10.1038/srep36633 (2016).

**Publisher’s note:** Springer Nature remains neutral with regard to jurisdictional claims in published maps and institutional affiliations.

## Figures and Tables

**Table 1:** Average working memory capacity (WMC; K-value), hand direction during baseline (HD-base; degrees), hand direction during washout (HD-wash; degrees), reaction time during baseline (RT-base; degrees), reaction time during adaptation (RT-adapt; degrees), movement time during baseline (MT-base; seconds), and movement time during adaptation (MT-adapt; seconds).

	Experiment 1 (N = 30)	Experiment 2 (N = 34)	Experiment 3 (N = 20)
WMC	1.99 ± 0.21	2.54 ± 0.21	1.84 ± 0.25
HD-base	−0.68 ± 0.33	−0.82 ± 0.21	−0.61 ± 0.25
HD-wash	2.88 ± 0.52	3.98 ± 0.44	24.44 ± 0.96
RT-base	0.44 ± 0.01	0.48 ± 0.02	0.39 ± 0.01
RT-adapt	1.46 ± 0.10	0.67 ± 0.04	0.55 ± 0.02
MT-base	0.37 ± 0.05	0.42 ± 0.06	0.33 ± 0.02
MT-adapt	0.62 ± 0.07	0.33 ± 0.03	0.30 ± 0.02

Mean ± SEM.
